# A pathogenic variant in the *FLCN* gene presenting with pure dementia: is autophagy at the intersection between neurodegeneration and cancer?

**DOI:** 10.3389/fnins.2023.1304080

**Published:** 2024-01-05

**Authors:** Irene Bottillo, Luigi Laino, Alessia Azzarà, Carla Lintas, Ilaria Cassano, Vincenzo Di Lazzaro, Francesca Ursini, Francesco Motolese, Simone Bargiacchi, Daniela Formicola, Paola Grammatico, Fiorella Gurrieri

**Affiliations:** ^1^Division of Medical Genetics, Department of Experimental Medicine, San Camillo-Forlanini Hospital, Sapienza University, Rome, Italy; ^2^Research Unit of Medical Genetics, Department of Medicine and Surgery, Università Campus Bio-Medico di Roma, Rome, Italy; ^3^Department of Medicine and Surgery, Unit of Neurology, Neurophysiology, Neurobiology and Psychiatry, Università Campus Bio-Medico di Roma, Rome, Italy; ^4^Unit of Neurology, Fondazione Policlinico Universitario Campus Bio-Medico, Rome, Italy; ^5^Operative Research Unit of Medical Genetics, Fondazione Policlinico Universitario Campus Bio-Medico, Rome, Italy

**Keywords:** FLCN, folliculin, Birt–Hogg–Dubé (BHD), dementia, autophagy, methionine

## Abstract

**Introduction:**

Folliculin, encoded by *FLCN* gene, plays a role in the mTORC1 autophagy cascade and its alterations are responsible for the Birt–Hogg–Dubé (BHD) syndrome, characterized by follicle hamartomas, kidney tumors and pneumothorax.

**Patient and results:**

We report a 74-years-old woman diagnosed with dementia and carrying a *FLCN* alteration in absence of any sign of BHD. She also carried an alteration of *MAT1A* gene, which is also implicated in the regulation of mTORC1.

**Discussion:**

The *MAT1A* variant could have prevented the development of a *FLCN*-related oncological phenotype. Conversely, our patient presented with dementia that, to date, has yet to be documented in BHD. Folliculin belongs to the DENN family proteins, which includes C9orf72 whose alteration has been associated to neurodegeneration. The folliculin perturbation could affect the C9orf72 activity and our patient could represent the first human model of a relationship between FLCN and C9orf72 across the path of autophagy.

## Introduction

Autophagy is a key homeostatic pathway that facilitates the degradation and recycling of cellular material, such as defective organelles and aggregates of misfolded protein, through lysosomes (Klionsky et al., 2021). It is directly involved in the regulation of development programs, the maintenance of the self-renewal potential of stem cells, cell differentiation and plasticity (Klionsky et al., 2021). Several components of the autophagic machinery have been found mutated in human diseases. In neurodegenerative conditions, defective autophagy impairs the removal of protein aggregates contributing to neurodegeneration onset and progression. Conversely, proficient autophagic responses support tumor progression and resistance to therapy (Klionsky et al., 2021) even though, in cancer, the role of autophagy appears to be more complex and depends on tumor stage, biology and the surrounding microenvironment (Debnath et al., 2023).

**FIGURE 1:**
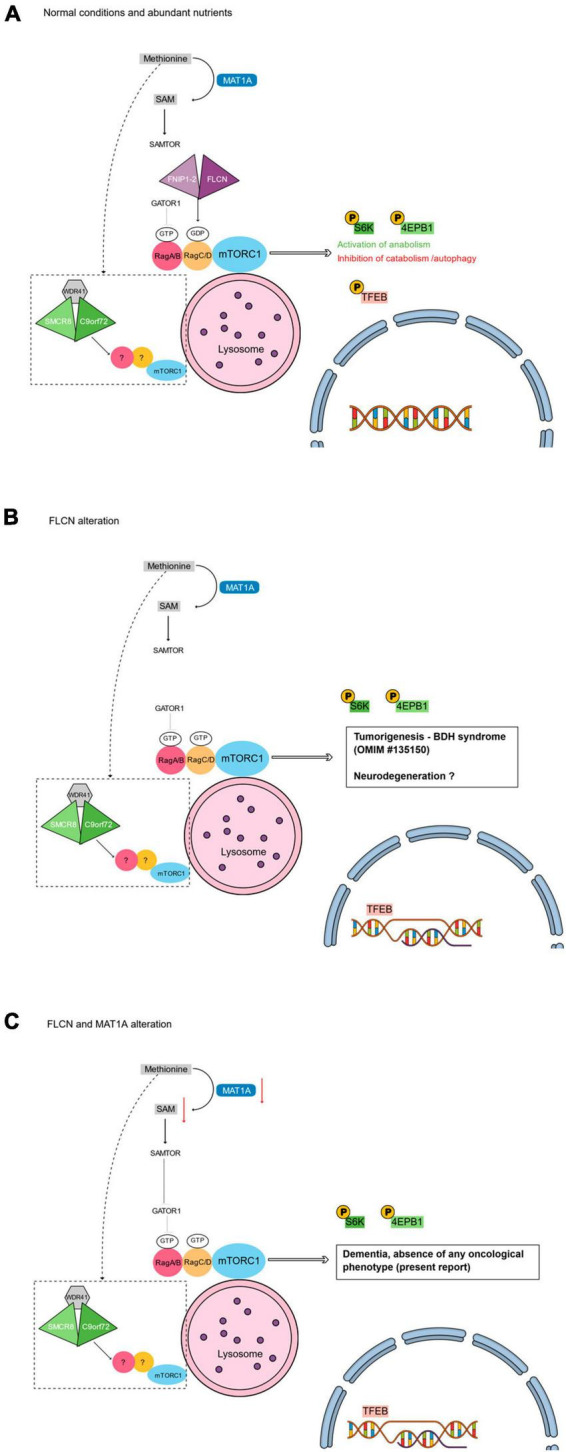
Main tiers of mTORC1 signaling that could intersect autophagy with the phenotype of the reported patient. **(A)** Under normal conditions, the presence of abundant nutrients (e.g., methionine) leads to the activation of mTORC1 pathway. The FLCN/FNIP-2 and the GATOR1 complexes activate the RagA/B-RagC/D dimer. The active RagA/B-RagC/D dimer enables the activation of mTORC1 at the lysosomal surface leading to the phosphorylation of different substrates. Phosphorylated S6K and 4EPB1 promote the synthesis of subcellular structures (e.g., proteins, lipids, and nucleotides), while the phosphorylation of TFEB prevents its translocation into the nucleus and inhibits autophagy (Napolitano et al., 2022). **(B)** Loss of function alterations of FLCN cause the inactivation of the RagC/D monomer that leads to the non-phosphorylation of TFEB. TFEB can then constitutively localize into the nucleus, where it induces the transcriptional program of autophagy and enhances the RagC/D expression, reflecting in an increased mTORC1 activity. The absence of FLCN does not interfere with the RagA/B activity, with a final effect of hyperphosphorylation of S6K and 4EBP1 (Napolitano et al., 2022). **(C)** Coupled with the absence of FLCN, the alteration of MAT1A may lead to a reduced level of SAM. SAMTOR would then sense the SAM deprivation and inhibit mTORC1 activity via GATOR1 binding. The resulting de-regulated autophagy could be responsible for the absence of any oncological phenotype in our patient. **(A–C)** *Dotted box*: In parallel with FLCN/FNIP1-2 and GATOR1 complexes, the complex formed by C9orf72, SMCR8, and WDR41 is supposed to act at the lysosome surface regulating mTORC1 (Amick and Ferguson, 2017).

The primary role of autophagy is to equip cells with the ability to maintain viability under nutrient-restricted conditions (Galluzzi et al., 2014). Indeed, being a degradative organelle, the lysosome is a key source of cellular metabolites, mostly in starvation. Hence, cells have the ability to sense different nutrient states for adapting to various environments and increase or decrease the nutrient availability (Sung et al., 2023): such nutrient sensing is regulated by different proteins acting in different pathways (Napolitano et al., 2022). Among these, the mechanistic target of rapamycin complex 1 (mTORC1 complex) is an important effector that integrates nutrient signaling with cell growth and metabolism (Seibert et al., 2021); its activity is modulated by sensors of nutrients’, in particular amino acids’, levels (Sung et al., 2023). mTORC1 activation occurs at the lysosomal surface and is dependent on the activity of Rag GTPase heterodimeric complexes which are composed of RagA or B (RagA/B) bound to RagC or D (RagC/D) (Sung et al., 2023). Two GTPase-activating protein (GAP) complexes, FLCN/FNIP1-2 (folliculin/folliculin-interacting protein 1/2) and GATOR1, convert the Rag GTPases between two stable states. The presence of abundant nutrients stimulates the GAP activity of FLCN/FNIP1-2 and of GATOR1 toward the Rag GTPases (Napolitano et al., 2022). Indeed, under high-nutrient conditions, the GATOR1 and the FLCN/FNIP-2 complexes mediate the exchange between, respectively, GDP/GTP on RagA/B, and GTP/GDP on RagC/D, and thus activate the dimer, that presents in the following state: RagA or B*^GTP^*/RagC or D*^GDP^* (Napolitano et al., 2022). This active dimer enables and sustains the activation of mTORC1 at the lysosomal surface (Napolitano et al., 2022; Figure 1A).

Different substrates are phosphorylated by active mTORC1, and this phosphorylation can be both activating and inhibitory. In particular, mTORC1 phosphorylates some substrates, including S6K and 4EPB1, that promote the synthesis of subcellular structures (e.g., proteins, lipids, and nucleotides), and some others, including ULK1 and the transcription factor EB (TFEB), that inhibit the catabolic processes. Phosphorylation prevents TFEB from translocating into the nucleus and activating genes related to autophagy and lysosome biogenesis. In summary, mTORC1 is active during high-nutrient conditions and it positively influences cellular anabolism while also preventing autophagy (Napolitano et al., 2022; Figure 1A).

Conversely, under low-nutrient conditions, the dimer RagA/B-RagC/D is inactive since RagA/B turns GDP-bound and RagC/D turns GTP-bound (i.e., under low-nutrient conditions, the dimer presents in the following state: RagA or B*^GDP^*/RagC or D*^GTP^*). The inactive RagA/B-RagC/D dimer involves the inactivation of mTORC1 (Napolitano et al., 2022). One of the effects of the mTORC1 inactivation is the non-phosphorylation of TFEB, that now can translocate into the nucleus and can activate the transcription of genes for autophagy, for lysosomal biosynthesis (Napolitano et al., 2022).

Constitutional loss-of-function variants of the tumor suppressor *FLCN* gene are responsible for the Birt–Hogg–Dubé (BHD) syndrome, an autosomal dominant disorder characterized by hair follicle hamartomas, kidney tumors, and spontaneous pneumothorax (Schmidt and Linehan, 2015). FLCN specifically mediates the activation of RagC/D that is crucial for the phosphorylation of TFEB. Therefore, in the absence of FLCN, as in BHD patients, TFEB is negatively regulated since non-phosphorylated; hence, it translocates to the nucleus to induce the transcriptional programs promoting autophagy and, also, to further promote mTORC1 activity through a negative feedback loop that relies on transcriptional induction of RagC/D GTPases (Napolitano et al., 2020). Such induction in the absence of FLCN results in an enhanced lysosomal recruitment of mTORC1 but not in an enhanced phosphorylation of TFEB. These findings explain the “paradoxical” hyperactivation of mTORC1 in BHD syndrome whereas TFEB is dephosphorylated because of loss of FLCN function (Napolitano et al., 2022; Figure 1B). The constitutive activation (i.e., dephosphorylation) of TFEB and subsequent amplification of its transcriptional program, is the main oncogenic driver of the kidney abnormalities in a mouse model of BHD syndrome, while TFEB depletion rescues renal pathology and lethality in *FLCN*-knockout mice (Napolitano et al., 2020). Moreover, mTORC1 signaling is hyperactive in up to 80% of human cancers (Menon and Manning, 2008). Because tumor microenvironments are poorly vascularized and subject to severe nutritional restrictions, loss of the mTORC1 nutrient sensing machinery may help cancer cells evade metabolic checks on anabolism and proliferation.

Genetic evidence supports a possible role of mTORC1 in several neurodegenerative disorders, such as Alzheimer’s disease (AD), Lafora’s disease, frontotemporal dementia (FTD), amyotrophic lateral sclerosis (ALS) and Parkinson’s disease, in which defects arise at the stage of substrate sequestration and of autophagosome formation (Nixon, 2013). Indeed, failures in the autophagic clearance have emerged as a key hallmark of neurotoxic cell death and, in mouse, the deletion of essential autophagy genes in the brain results in neurodegeneration with behavioral defects, including abnormal limb-clasping reflexes and a reduction in coordinated movement (Komatsu et al., 2006). Impaired autophagy is also involved in the accumulation of abnormal protein aggregates, including amyloid plaques [composed of beta-amyloid (Aβ) peptides] and neurofibrillary tangles (formed by hyper-phosphorylated tau protein), at the basis of AD (Zhang et al., 2021).

## Report

We here report on a 74-years-old woman diagnosed with dementia, whose clinical and molecular findings led us to speculate about a possible link between neurodegeneration, autophagy and cancer. Her comorbidities were essential hypertension, dyslipidemia and chronic bronchitis. She smoked 40 cigarettes a day for more than three decades. About 1 year before her first neurological evaluation, at the age of 72 years old, she insidiously started to present an impairment in short-term memory, impaired ability to acquire and remember new information, which got worse over time. She felt uncertain, anxious, and concerned about her memory impairment. She became more neglected in personal care and developed social withdrawal. She got into difficulties in cooking and consequently lost weight, and also made some mistakes in the assumption of her usual therapy.

**FIGURE 2:**
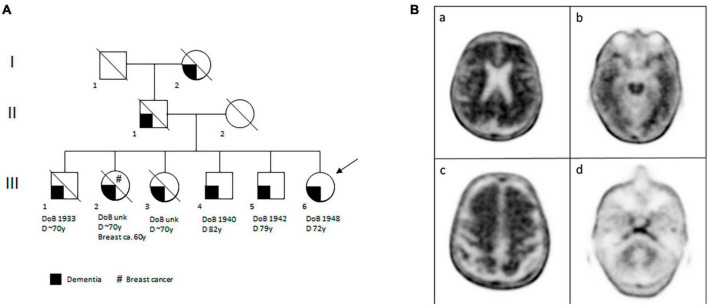
The proband’s familial and brain features. **(A)** Pedigree of the proband’s family. The clinical legend is given under the pedigree. DoB, date of birth; D, dementia; Breast ca., breast cancer; y, years; unk, unknown. **(B)** Amyloid PET with [18F]-florbetaben performed in patient III:6 and showing diffuse amyloid deposition, in particular in the brain’s posterior cingulate cortex, lateral parietal (panel a) and temporal cortices (especially regarding the left lateral temporal cortex, panel b). Amyloid deposition is also seen in the frontal region (panel c). Panel d shows radiotracer uptake in the cerebellum, as reference.

The family history was positive for dementia in two sisters, three brothers, the father and the paternal grandmother (Figure 2A). The first three siblings (one brother and two sisters) developed cognitive deficits when they were around 70 years old and, afterward, cognitive impairment became striking around 5 years later. The deficits were constituted above all by impairment of short memory, space-time disorientation, and in the first brother also by a deficit of language. One of the two sisters began with depression and, after that, she developed dementia; in addition, she was also diagnosed with breast cancer at 60 years old. Every one of these siblings is already dead. The other two brothers are still alive: one is 83 years old and he developed memory and language impairment 1 year ago. The other brother is 81 years old, he has had dementia for 2 years with a more aggressive course than the others. The last sibling is our case patient.

On neurological evaluation, our patient appeared sufficiently oriented in time and space, the examination did not detect any neurological focal deficit and there were no extrapyramidal signs. The initial Mini Mental Status Examination (MMSE) (Folstein et al., 1975) was 26/30, at the lower limit of normal range. Blood tests were normal, except for hypercholesterolemia. Thyroid function and levels of folic acid and B12 vitamin were in the normal range. Brain magnetic resonance imaging showed punctiform gliotic areas in white matter, enlargement of supratentorial ventricular system and of cortical subarachnoid spaces. A second level neuropsychological examination was performed, which revealed impairment of episodic memory with difficulties in learning and recall of recently learned information, deficit in visual memory, reduction of visuoconstructive skills and troubles in setting shifting.

The patient refused to undergo lumbar puncture for the dosage of cerebrospinal fluid Aβ (CSF Aβ42) and phospho-tau, so an amyloid-PET imaging with 18F-florbetaben was performed. It resulted positive for amyloid plaques (brain amyloid plaque load = 3) (Figure 2B). According to the 2018 NIA-AA criteria, a diagnosis of Alzheimer dementia was made and donepezil 10 mg/die was prescribed. A slow progressive cognitive impairment of the patient was observed in the five subsequent neurological evaluations. The last MMSE score, 18 months after her first evaluation, was 22/30 (indicating mild to moderate dementia).

According to her family history positive for dementia, the patient was referred to genetic counseling and genetic testing. Whole exome sequencing (WES) was performed on genomic DNA extracted from peripheral blood through a Nextera DNA Exome kit (Illumina, San Diego, CA, USA) using a NextSeq2000 sequencer (Illumina). Sequencing reads were aligned to the human reference genome (UCSC hg19) by the BWA software package (v0.7.7-isis-1.0.2) (Illumina). Variant calling was performed by the GATK Variant Caller (v1.6-23-gf0210b3). The DNA variants were annotated by eVai (v.2.7) (EnGenome). After filtering by MAF < 0.01 (GnomAD v2.1), variants mapping in genes associated to the following human phenotype ontology (HPO) phenotypes were prioritized: HP:0000726 (dementia), HP:0000727 (frontal lobe dementia), HP:0001268 (mental deterioration), HP:0002145 (FTD), HP:0002439 (frontolimbic dementia), HP:0002511 (AD), HP:0007123 (subcortical dementia), HP:0030219 (semantic dementia), HP:0033051 (impaired executive functioning). Filtered variants were classified according to ACMG/AMP criteria (Richards et al., 2015). No pathogenic or likely pathogenic variants were identified in the genes associated with the selected HPO phenotypes. Moreover, the APOE e4 pathogenic allele were not present in the patient. However, subsequent filtering for rare alterations resulted in the identification of a heterozygous pathogenic variant in the folliculin gene: the *FLCN* NM_144997.7:c.890_893delAAAG (p.Glu297AlafsTer25). The presence of this variant was confirmed by Sanger sequencing (Supplementary Figure 1A). This frameshift variant showed an extremely low frequency in the GnomAD population database (MAF = 0.000795%) and it was predicted to cause the Nonsense Mediated Decay (NMD) of the transcript, thus leading to haploinsufficiency. Also, the c.890_893delAAAG was already reported as pathogenic both in ClinVar database and in patients affected by BHD syndrome (Woodward et al., 2008; Kluger et al., 2010; Dow and Winship, 2016; Sprague and Landau, 2016).

## Discussion

The penetrance of BHD syndrome is considered to be very high: approximately 90%–95% of individuals with a heterozygous germline *FLCN* pathogenic variant develop at least one feature of the syndrome (Sattler and Steinlein, 1993). Conversely, our 74-years-old proband, did not show any of the clinical signs of the syndrome, suggesting the possible presence of a modifier factor for the disease. Unfortunately, as all family members were deceased or unavailable, we could not perform the segregation analysis of the identified DNA variants.

An emerging aspect of cancer metabolism is the role of essential amino acid methionine, the most variable metabolite present in human plasma. One of the most prominent functions of methionine is its contribution to intracellular methylation by serving as the sole source of the universal methyl donor S-adenosyl-methionine (SAM) (Gu et al., 2017). Indeed, the methionine adenosyltransferase (MAT) converts methionine into SAM, that is a substrate for all the methylation reactions of the cell, including those that modulate gene expression (Sanderson et al., 2019). Moreover, several cytosolic amino acid sensors, such as SAMTOR (S-adenosylmethionine sensor upstream of mTORC1), sense cytosolic SAM levels and convey this information to mTORC1 (Gu et al., 2017). Upon SAM deprivation, as a marker for the intracellular deprivation of methionine, SAMTOR inhibits mTORC1 activity (Gu et al., 2017). Conversely, as previously described, an increase of amino acid levels, leads to the activation of mTORC1 which inhibits autophagy by phosphorylating TFEB and the other regulators of cellular anabolism, lysosomal biogenesis and autophagy (Martina et al., 2014; Napolitano et al., 2022).

Dietary restriction of methionine (MR) influences cell behavior and cancer outcome through controlled changes to methionine metabolism. Indeed, MR was shown to produce therapeutic responses in chemoresistant colorectal cancer patient derived xenografts (Gao et al., 2019). Moreover, tumor-initiating cells exhibit elevated activity of methionine metabolism’s enzymes, most notably an upregulation of MAT expression and activity (Wang et al., 2019). As a results of this emerging evidence, numerous clinical trials investigating compounds that directly or indirectly target methionine metabolism have been set up on different cancer types (Sanderson et al., 2019).

Humans possess two genes encoding isoforms of MAT: *MAT1A* gene that encodes a catalytic subunit that forms the tetrameric and dimeric holoenzymes, MAT I and MAT III, and *MAT2A* gene that encodes the catalytic subunit of MAT II (Kotb et al., 1997). MAT II is the major isozyme in non-hepatic tissues and in fetal liver, whereas MAT I and III are the major forms present in post-natal liver (Kotb et al., 1997). While to date no human models of *MAT2A* pathogenic mutations have been described (OMIM *601468), *MAT1A* alterations have been associated to MAT deficiency (OMIM #250850). Patients with this deficiency develop hypermethioninemia. MAT I/III deficiency can be inherited as autosomal dominant or as recessive traits. Most patients have no clinical abnormalities, although some with the autosomal recessive forms may show neurologic abnormalities (Kim et al., 2016).

The discovery that the proband also had a heterozygous pathogenic variant in the gene encoding the MAT was surprising: she indeed was found to carry the *MAT1A* NM_000429.3:c.596G>A (p.Arg199His) variant. The presence of this variant was confirmed by Sanger sequencing (Supplementary Figure 1B). The *MAT1A*:c.596G>A missense variant, showing a low frequency in GnomAD population database (i.e., MAF = 0.0016%), was predicted to affect protein structure/function (i.e., revel score: 0.884), was reported as pathogenic in ClinVar database, and mapped in a codon for which a different amino acid change was reported as pathogenic in ClinVar. Additionally, the c.596G>A had already been observed in at least one individual with autosomal recessive MAT I/III deficiency (Chien et al., 2015). Also, by expressing the mutated allele in bacteria cells, the relative activity of the p.Arg199His isoform has been proven to be about 11% of wild-type MAT activity (Chamberlin et al., 1996), although the c.596G>A carriers do not usually display hypermethioninemia (Chien et al., 2015). Along this line, in our patient, most amino acids’ levels in peripheral blood were within the normal range. In particular, methionine concentration was 26 μM/L (normal range: 10–50μM/L) (Supplementary Figure 1C).

Interestingly, in our patient, the absence of any oncological phenotype actually reflects the absence of the cells proliferative phenotype that would be instead expected from the presence of the *FLCN*:c.890_893delAAAG loss-of-function alteration. We hypothesize that the MAT1A:p.Arg199His isoform may actually lead to a reduced level of SAM, and consequently to a control of cell proliferation and behavior, as observed in the models of methionine dietary restriction (Gao et al., 2019; Figure 1C).

Conversely, our patient presented with dementia, a clinical feature that, to date, has never been reported in patients carrying a pathogenic *FLCN* variant. But interestingly, Nookala et al. (2012) did reveal that the crystal structure for the C-terminal fragment of FLCN, contained a DENN (differentially expressed in normal and neoplastic cells) domain that was highly similar to the DENN domain of the C9orf72 protein (Nookala et al., 2012), whose dominant expansion of a noncoding GGGGCC hexanucleotide repeat has been proven to be causative of both ALS and FTD (DeJesus-Hernandez et al., 2011). Moreover, C9orf72 repeat expansion may play a role in the neurodegeneration associated to AD (Lall et al., 2021; Vinceti et al., 2023).

Differentially expressed in normal and neoplastic cells domain proteins are highly conserved GDP/GTP exchange factors (GEFs) that activates Rab-GTPases, with large numbers of homologues in some species (Levine et al., 2013). From a structural point of view, C9orf72 was also predicted to have closest structural similarity to key FLCN-binding partners, FNIP1/2, that are also members of the DENN family (Pacitto et al., 2015). Therefore, collectively, both C9orf72, FLCN and FNIP1-2 proteins contain a DENN domain. More importantly, as the FLCN/FNIP1-2 complex interacts with the Rags for regulating autophagy in response to amino acids’ abundance, likewise C9orf72 functions at lysosomes as part of a larger complex that also contains other DENN proteins (i.e., the Smith-Magenis chromosome region 8, SMCR8, and WD repeat-containing protein 41, WDR41). Also, in parallel with FLCN, such activities of C9orf72 might be tightly regulated by nutrient availability (Amick and Ferguson, 2017). No interactions have been yet detected for C9orf72 with either FLCN and FNIP1-2, indicating that there is specificity for the formation of two distinct complexes within this subfamily of DENN domain proteins: the FLCN/FNIP1-2 complex and the C9orf72/SMCR8/WDR41 complex (Amick et al., 2016). The structural orientation of the C9orf72/SMCR8/WDR41 complex is also reminiscent of FLCN/FNIP1-2, which suggested it might possess GAP activity (Jansen and Hurley, 2023). Then, these two different protein ensembles act at lysosomes with functions that seem related but distinct and that have yet to be elucidated (Amick and Ferguson, 2017; Jansen and Hurley, 2023; Figure 1C). Moreover, most *FLCN* mutations isolated from BHD patients are loss of function leading to decreased protein stability or inability to interact with FNIP1/2 (Schmidt and Linehan, 2018), likewise the C9orf72 repeat expansion found in ALS and dementia cases results in a loss of function due to reduced C9orf72 protein expression (Waite et al., 2014).

Based on these evidence that collectively highlight both structural similarities and parallel functions of the FLCN/FNIP1-2 and C9orf72/SMCR8/WDR41 complexes, and based on the neurodegenerative pathology of our proband, we hypothesize that a perturbation of the FLCN/FNIP1-2 (i.e., the FLCN loss of function alteration) might produce a perturbation of the C9orf72/SMCR8/WDR41. In this view, neurodegenerative disorders could be present but to date undiagnosed in BHD patients. The here presented patient could provide the rationale for retrospective and perspective studies on large BHD cohorts of patients, with the aim of evaluating the presence of neurodegenerative phenotypes and, if present, their penetrance.

## Conclusion

In conclusion, the present patient could represent the first demonstration in humans of a genetic interaction between *FLCN* and C9orf72 across the path of autophagy. Both the oncological phenotype observed in *FLCN* mutated patients and the neurodegeneration observed in C9orf72 mutated cases, might indeed be the result of the dysregulation of mTORC1 signaling. As in reverse genetics approaches, further studies that might reveal the interactions between the proteins constituent of the FLCN/FNIP1-2 and the C9orf72/SMCR8/WDR41, could support our observation. Moreover, the absence of any oncological disease in our case might be the first evidence in humans that the modulation of the autophagy pathway by the restriction of SAM can repress cell proliferation. Finally, as mTORC1 now reasonably attracts much attention owing to its central role in the control of cell metabolism in response to nutrients’ levels, future studies on proband’s cell lines and on animal models may further establish principles of how changes in amino acids levels (e.g., by dietary interventions) may be used to influence cellular behavior and fate, both in cancer and in neurodegeneration.

## Data availability statement

The original contributions presented in this study are included in this article/Supplementary material, further inquiries can be directed to the corresponding author.

## Ethics statement

The studies involving humans were approved by Ethics Committee of the Catholic University of the Sacred Heart of Rome. The studies were conducted in accordance with the local legislation and institutional requirements. The participants provided their written informed consent to participate in this study. Written informed consent was obtained from the individual(s) for the publication of any potentially identifiable images or data included in this article.

## Author contributions

IB: Conceptualization, Data curation, Formal analysis, Investigation, Methodology, Writing – original draft, Writing – review & editing. LL: Investigation, Methodology, Writing – review & editing, Validation. AA: Investigation, Methodology, Writing – review & editing, Formal analysis, Visualization. CL: Formal analysis, Investigation, Methodology, Visualization, Writing – review & editing. IC: Formal analysis, Investigation, Visualization, Writing – review & editing. VD: Conceptualization, Resources, Supervision, Writing – review & editing. FU: Data curation, Investigation, Writing – review & editing. FM: Data curation, Investigation, Methodology, Writing – review & editing. SB: Data curation, Formal analysis, Investigation, Methodology, Writing – review & editing. DF: Data curation, Formal analysis, Investigation, Methodology, Writing – review & editing. PG: Funding acquisition, Project administration, Resources, Supervision, Validation, Writing – review & editing. FG: Conceptualization, Data curation, Investigation, Supervision, Validation, Visualization, Writing – review & editing.

## Supplementary material

Supplementary Figure 1 The results of the proband’s molecular analysis. Sanger sequencing electropherograms showing the presence, in the proband, of two heterozygous variants: the NM_144997.7:c.890_893delAAAG (p.Glu297AlafsTer25) in *FLCN* **(A)** and the NM_000429.3:c.596G>A (p.Arg199His) in *MAT1A* **(B)**. **(C)** Plasma levels of amino acids.
