# Supplementary figures and images for: A pathogenic variant in the FLCN gene presenting with pure dementia: is autophagy at the intersection between neurodegeneration and cancer?
Source: Front Neurosci. 2024 Jan 5;17:1304080. doi: 10.3389/fnins.2023.1304080 (PMC10796570; doi:10.3389/fnins.2023.1304080)

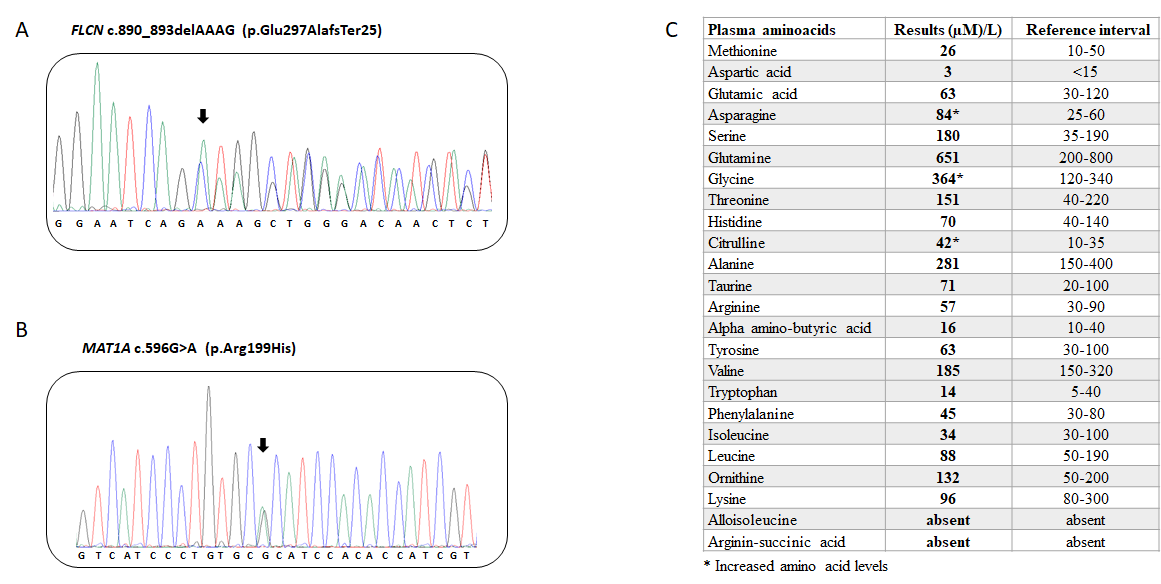

Supplement: Supplementary Figure 1 — The results of the proband’s molecular analysis. Sanger sequencing electropherograms showing the presence, in the proband, of two heterozygous variants: the NM_144997.7:c.890_893delAAAG (p.Glu297AlafsTer25) in FLCN (A) and the NM_000429.3:c.596G>A (p.Arg199His) in MAT1A (B). (C) Plasma levels of amino acids. [file Image_1.TIF]
